# Trastuzumab-induced upregulation of a protein set in extracellular vesicles emitted by ErbB2-positive breast cancer cells correlates with their trastuzumab sensitivity

**DOI:** 10.1186/s13058-020-01342-2

**Published:** 2020-10-06

**Authors:** Arik Durcker, Byong Hoon Yoo, Iman Aftab Khan, Dongsic Choi, Laura Montermini, Xiaoyang Liu, Sanja Jovanovic, Tallal Younis, Kirill V. Rosen

**Affiliations:** 1 Department of Medicine, Dalhousie University, Halifax, NS Canada; 2 Departments of Pediatrics & Biochemistry and Molecular Biology, Dalhousie University, Halifax, NS Canada; 3 Research Institute of the McGill University Health Centre, Glen Site, McGill University, Montreal, QC Canada; 4 Atlantic Research Centre, Rm C-304, CRC, 5849 University Avenue, PO Box 15000, Halifax, NS B3H 4R2 Canada

**Keywords:** Breast cancer, ErbB2, HER2, Trastuzumab, Extracellular vesicles, Biomarkers

## Abstract

**Background:**

ErbB2/HER2 oncoprotein often drives breast cancers (BCs) which are treated with the anti-ErbB2 antibody trastuzumab. The efficacy of trastuzumab-based metastatic BC therapies is routinely assessed by imaging studies. Trastuzumab typically becomes ineffective in the case of this disease and is then replaced by other drugs. Biomarkers of BC trastuzumab response could allow imaging studies and the switch to other drugs to occur earlier than is now possible. Moreover, bone-only BC metastases can be hard to measure, and biomarkers of their trastuzumab response could facilitate further treatment decisions. Such biomarkers are presently unavailable. In this study, we searched for proteins whose levels in BC cell-emitted extracellular vesicles (EVs) potentially correlate with BC trastuzumab sensitivity.

**Methods:**

We isolated EVs from cultured trastuzumab-sensitive and trastuzumab-resistant human BC cells before and after trastuzumab treatment and characterized these EVs by nanoparticle tracking analysis and electron microscopy. We found previously that ErbB2 drives BC by downregulating a pro-apoptotic protein PERP. We now tested whether trastuzumab-induced PERP upregulation in EVs emitted by cultured human BC cells correlates with their trastuzumab sensitivity. We also used mass spectrometry to search for additional proteins whose levels in such EVs reflect BC cell trastuzumab sensitivity. Once we identified proteins whose EV levels correlate with this sensitivity in culture, we explored the feasibility of testing whether their levels in the blood EVs of trastuzumab-treated metastatic BC patients correlate with patients’ response to trastuzumab-based treatments.

**Results:**

We found that neither trastuzumab nor acquisition of trastuzumab resistance by BC cells affects the size or morphology of EVs emitted by cultured BC cells. We established that EV levels of proteins PERP, GNAS2, GNA13, ITB1, and RAB10 correlate with BC cell trastuzumab response. Moreover, these proteins were upregulated during trastuzumab-based therapies in the blood EVs of a pilot cohort of metastatic BC patients that benefited from these therapies but not in those derived from patients that failed such treatments.

**Conclusions:**

Upregulation of a protein set in EVs derived from cultured breast tumor cells correlates with tumor cell trastuzumab sensitivity. It is feasible to further evaluate these proteins as biomarkers of metastatic BC trastuzumab response.

## Introduction

Approximately 20% of breast tumors overproduce ErbB2/Her2 receptor tyrosine kinase [1]. ErbB2 drives these cancers [1], and they are often treated with ErbB2 neutralizing anti-ErbB2 antibodies trastuzumab and pertuzumab [2]. Metastatic breast cancers typically develop resistance to these drugs [3]. Median progression-free survival of patients with metastases receiving trastuzumab and chemotherapy is approximately 18 months [3]. Response to these therapies is normally assessed by imaging studies (e.g., CT and/or bone scan) every 1.5–6 months, depending on the clinical scenario, e.g., treatment regimen or disease burden [4]. When tumors progress, the standard of care is to switch the patient to the “second-line” drug T-DM1, a conjugate between trastuzumab and a chemotherapeutic agent DM1, that acts by mechanisms distinct from those of trastuzumab [5]. The trastuzumab moiety of T-DM1 binds ErbB2 on the tumor cell surface (this likely does not require ErbB2 to be active), T-DM1 is internalized by the cells, trastuzumab is degraded, and DM1 kills the cells [5].

Trastuzumab is costly [6] and can have serious side effects (e.g., cardiotoxicity [7]). Access to a marker indicating that trastuzumab-based therapy is ineffective could allow oncologists to verify this lack of efficacy by imaging studies earlier than planned and switch patients to T-DM1 sooner or perform the switch without waiting for imaging studies. The patient could then stop receiving the ineffective and costly drug earlier than is now possible, avoid its side effects, and benefit from the timely switch to T-DM1 that is often successfully used to attenuate trastuzumab-resistant cancers [5]. The early switch could be especially critical for patients with high-volume disease and/or significant visceral metastasis when the patient may have months to live and the time frame for treatment decisions is limited. Another unmet need in the biomarkers of trastuzumab efficacy relates to the fact that ErbB2-positive breast tumors often metastasize to the bone [8]. Trastuzumab response in blastic or mixed blastic/lytic bone-only metastases is often difficult to measure by imaging studies [9]. Hence, the availability of a marker indicating whether the lesion responds to trastuzumab could strongly facilitate further treatment decisions. Reliable biomarkers of breast cancer trastuzumab sensitivity are now unavailable [4].

All cell types emit extracellular vesicles (EVs) [10]. EV biogenesis mechanisms are diverse. For example, EVs called exosomes (50–150 nm in size) are released by cells as a result of fusion of multivesicular endosomes with the plasma membrane [10], while EVs called ectosomes (100–1000 nm in size) are emitted by cells via the plasma membrane budding. EV proteins, such as TSG101, are exosome markers, while other proteins, e.g., Flotillin-1, are markers of exosomes and other EV types [10].

Cellular proteins, RNA, and DNA are sorted into EVs by poorly understood mechanisms [10, 11]. EVs are present in the blood [10]. The content of tumor cell-emitted EVs often differs from that of EVs produced by the normal cells [12]. For example, blood EVs of breast cancer patients have higher levels of various proteins (e.g., EPCAM) [13] or RNAs (e.g., miR-372) [14] than EVs derived from the blood of healthy donors*.* Such EV components were proposed to be potential biomarkers that could serve for breast cancer detection [13, 14]*.* However, EV-based biomarkers of breast cancer trastuzumab sensitivity are not available.

In this study, we have identified proteins whose upregulation in breast tumor cell-emitted EVs reflect trastuzumab sensitivity of cultured breast tumor cells. Moreover, we found that upregulation of these proteins in EVs derived from the blood of a pilot cohort of breast cancer patients with metastatic ErbB2-positive breast cancer tends to correlate with trastuzumab sensitivity of patients’ tumors. Hence, these proteins can be studied further as potential biomarkers of breast cancer trastuzumab response.

## Methods

### Materials

Trastuzumab was from Roche, Mississauga, ON.

### Cell culture

BT-474 cells (American Type Culture Collection, Manassas, VA, USA) were cultured in Hybri-Care medium (American Type Culture Collection, Manassas, VA, USA), 10% fetal bovine serum (FBS), 100 U/ml penicillin (GIBCO, Waltham, MA, USA), 100 μg/ml streptomycin (GIBCO, Waltham, MA, USA), and 0.29 mg/ml l-glutamine (GIBCO, Waltham, MA, USA). HCC1419 (American Type Culture Collection, Manassas, VA, USA) cells were cultured in RPMI1640 medium (GIBCO, Waltham, MA, USA), 10% FBS (Sigma-Aldrich St. Louis, MO, USA), 100 U/ml penicillin (GIBCO Waltham, MA, USA), 100 μg/ml streptomycin (GIBCO, Waltham, MA, USA), and 0.29 mg/ml l-glutamine (GIBCO). MCF-7/Her2-18 cells were provided by Dr. Hung Mien-Chie (MD Anderson Cancer Center, Houston, TX, USA) and grown in Dulbecco’s modified Eagle’s medium/nutrient mixture F-12 Ham (Sigma Life Science) containing 10% fetal bovine serum and 1% penicillin–streptomycin. To generate BT474TR cells, 1 × 10^6^ BT-474 cells were cultured in suspension (above a layer of Sea Plaque agarose) for 2 weeks in the presence of 5 μg/ml trastuzumab. The surviving cells were then grown in the monolayer culture in the presence of 5 μg/ml trastuzumab for 4 months. Lack of mycoplasma contamination in the cells was established by the use of MycoFluor Mycoplasma Detection Kit (Molecular Probes, Eugene, OR, USA) according to the manufacturer’s instructions.

### Soft agar colony formation assay

Cells were suspended in 2 ml of their growth medium containing 0.3% of melted Bacto-agar. Cell suspensions were added to a 60-mm plate covered with a 2-ml layer of solidified 0.5% Bacto-agar polymerized in the same medium. Cell colonies were counted 7–10 days later.

### Isolation of EVs in tissue culture

Cells were grown above a layer of Sea Plaque agarose in all cases except for the electron microscopy studies where cells’ suspension culture was achieved by placing the cells in bacteriological dishes in the media containing 10% EV-depleted FBS. The cell medium was then subjected to centrifugation at 400*g* for 10 min, 2500*g* for 10 min, and 10,000*g* for 30 min. The resulting supernatant was subjected to ultracentrifugation at 100,000*g* for 1 h, and the pellet was resuspended in the phosphate-buffered saline (PBS) and further subjected to ultracentrifugation at 100,000*g* for 1 h.

### Isolation of EVs from patients’ blood

Ten microliters of blood was collected into the citrate/CTAD tube and mixed gently to ensure exposure to anticoagulant-coated walls. The plasma was separated by centrifugation of the blood sample at 2500*g* for 15 min at 4 °C. The clear top layer (plasma) was transferred to a labeled tube and stored at − 80 °C. To isolate EVs, the plasma was subjected to centrifugation at 10,000*g* for 30 min and the resulting supernatant was filtered through the 0.2-μm filter. The filtrate was subjected to ultracentrifugation at 100,000*g* for 1 h, and the pellet was resuspended in the phosphate-buffered saline and further subjected to ultracentrifugation at 100,000*g* for 1 h.

### Nanoparticle tracking analysis

The cells were grown in suspension on bacteriological dishes. The cell medium was subjected to centrifugation at 10,000*g* for 30 min followed by filtration through the 0.2-μm filter. Nanoparticle tracking analysis (NTA) was carried out using NanoSight NS500 instrument with a 532-nm laser (NanoSight Ltd., UK). Three recordings of 30 s at 37 °C in camera level 15 were obtained and processed using NTA software (version 3.0).

### Liquid chromatography-tandem mass spectrometry analyses

EVs isolated as described above were subjected to in-gel digestion within the presence of dithiothreitol and iodoacetic acid as previously described [15]. The eluted peptides were lyophilized and re-solubilized in 0.1% formic acid/2% acetonitrile. The peptides were loaded onto a Thermo Acclaim Pepmap (75-μm inner diameter × 2 cm with C18 beads) (Thermo Fisher Scientific, San Jose, CA) pre-column and onto the Acclaim Pepmap Easyspray (75-μm inner diameter × 15 cm with C18 beads) (Thermo Fisher Scientific) analytical column. Separation was achieved using a Dionex Ultimate 3000 uHPLC at 220 nl/min with a gradient of 2–35% organic solvents (0.1% formic acid in acetonitrile) for 3 h. Peptides were analyzed using a Thermo Orbitrap Fusion mass spectrometer operating at 120,000 resolution (FWHM in MS1, 15,000 for MS/MS) with higher-energy collisional dissociation sequencing all peptides with a charge of 2+ or greater. The raw data were converted into *.mgf format (Mascot generic format) by MSConvert (ProteinWizard) and analyzed by the Mascot 2.5.1 software against the SwissProt (http://www.uniprot.org) human protein database (release 2018_11, 20,413 entries). The tolerance was 5.0 ppm monoisotopic for the precursor ions and 0.100 Da for fragment ions. The permission of two potential missed cleavages was selected for trypsin digestion. Carboxymethyl of cysteine (58 Da) was specified as a fixed modification. Deamidation of asparagine (1 Da) and glutamine (1 Da) and oxidation of methionine (16 Da) were specified as variable modifications. The resulting data were further analyzed by the Scaffold Q+ software (version 4.8.8) (Proteome Sciences, Portland) (protein threshold > 0.95%, peptide threshold > 0.95%, and 2 of minimum number of unique peptides). We quantified the relative protein abundance from the data sets of each proteome, by normalizing the total ion count (TIC) using the Scaffold Q+ software, in which protein abundance was calculated by calculating the sum of the areas under all the peaks contained in the MS/MS spectra assigned to a protein.

### Transmission electron microscopy

A 10-μl drop of a PBS solution containing EVs was placed on the formvar/carbon-coated grid for 15 min followed by rinsing with a gentle stream of distilled water. The sample was then fixed with 2% uranyl acetate for 30 s. The images were captured by the use of the JEOL JEM 1230 instrument.

### Western blotting

The western blotting analysis was performed as previously published [16]. The following antibodies were used: anti-Her2 (Cell Signalling, catalogue #2242), anti-Flotillin-1 (Cell Signalling, catalogue #18634), anti-integrin beta 1 (Cell Signalling, catalogue #9699), anti-RAB10 (Cell Signalling, catalogue #8127), anti-PERP (Abcam, catalogue ab5986), anti-GNAS2 (Abcam catalogue #ab83735), and anti-GNA13 (catalogue #Abcam, ab128900). When lanes were removed from the western blot images, and separate parts of an image were joined together, a short vertical black line was used to indicate where the image was cut. Western blot signals were quantified using an Odyssey Imaging System-associated software v3.0 (LI-COR Biosciences) or the Quantity One software (Biorad).

### Clinical study

Patients with metastatic ErbB2-positive breast cancer were recruited in the QEII Health Centre, Halifax, NS, in 2018. The study was approved by the Research Ethics Board of Nova Scotia Health Authority (file #1022984). All study participants provided written informed consent. The study inclusion criteria were as follows. Patients (1) were female and over 18 years old, (2) had recurrent or newly diagnosed ErbB2-positive metastatic BC regardless of the hormone receptor status, (3) were recruited prior to starting or during trastuzumab-based therapy, and (4) were followed by medical oncologists. ErbB2-positive cancer was defined by standard immunohistochemistry and/or fluorescence in situ hybridization criteria [17]. The exclusion criteria were (1) another synchronous cancer or (2) history of prior cancer (except for non-melanoma or basal cell skin cancer) in the past 10 years. Patients’ lesions were assessed based on CT scans using the Response Evaluation Criteria in Solid Tumors version 1.1 [9].

### Statistical analysis

Statistical analysis of the data in Fig. 5 was performed by the chi-square test for goodness-of-fit. Statistical analysis of all other data was performed by Student’s *t* test.

## Results

### The size distribution of EVs emitted by ErbB2-positive breast cancer cells is not affected by trastuzumab or by the sensitivity of the cells to this drug

**Fig. 1:**
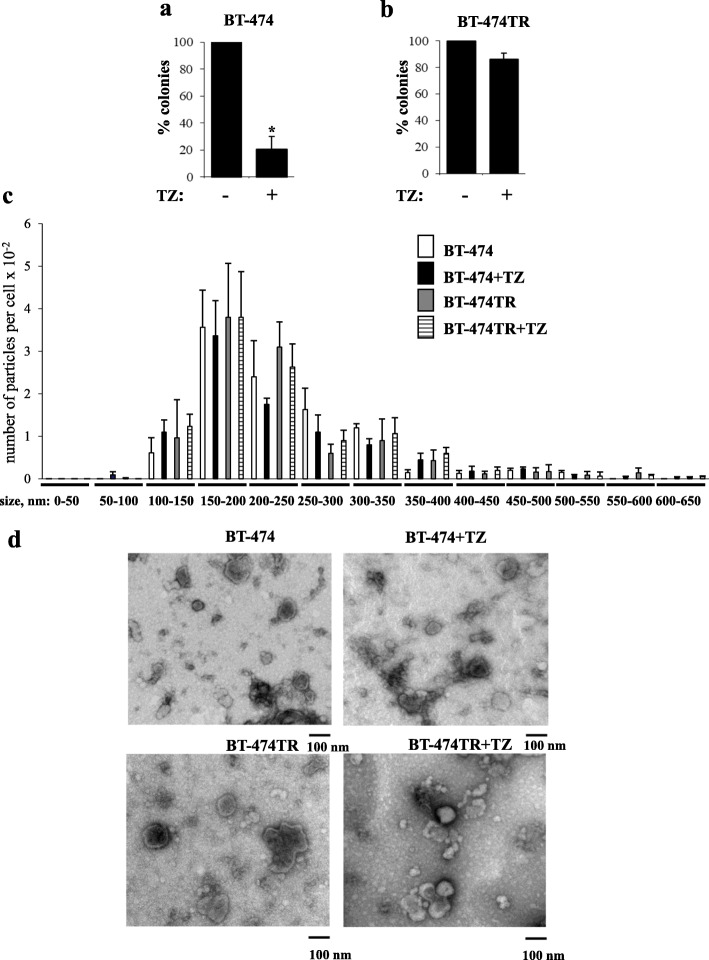
The number, size, and morphology of extracellular vesicles emitted by trastuzumab-sensitive and trastuzumab-resistant breast tumor cells are similar to each other and are not affected by trastuzumab. **a**, **b** Human breast cancer cells BT-474 (**a**) or their variant BT-474TR (**b**) were allowed to form colonies in soft agar in the absence (−) or in the presence (+) of 5 μg/ml trastuzumab (TZ). The colonies were counted 3 weeks later. The data represent the average of three independent experiments plus the SE.*p < 0.05. **c** BT-474 and BT-474TR cells were treated (+TZ) or not with 5 μg/ml trastuzumab in 3D culture for 72 h. The analysis of the nanoparticle size distribution in the cell media was performed by the use of the NS500 Nanoparticle Tracking Analysis system. The data represent the average of 3 independent experiments plus SD. Nanoparticle size distribution did not show a statistically significant difference between BT-474 and BT-474TR cells nor between trastuzumab-treated and untreated cells in the case of both cell lines. **d** BT-474 and BT-474TR cells were treated (+TZ) or not with 5 μg/ml trastuzumab in 3D culture for 72 h, and EVs were isolated from the cell media by ultracentrifugation and analyzed by transmission electron microscopy

To search for proteins whose EV levels correlate with breast tumor trastuzumab response, we generated a trastuzumab-resistant variant of ErbB2-positive breast cancer cells BT-474 [18] (BT-474TR) by exposing the cells in three-dimensional (3D) culture (when cells were detached from the extracellular matrix (ECM) [19]) to trastuzumab and expanding the surviving cells in the presence of the drug [19]. We used 3D culture in these and further studies since breast tumors and metastases grow in vivo as 3D masses [20]. 3D culture is thought to better mimic these conditions than the traditional monolayer culture [21]. As shown in Fig. 1a, the ability of the parental BT-474 cells to form colonies in the absence of adhesion to the ECM in soft agar was strongly reduced in the presence of trastuzumab. In contrast, trastuzumab had essentially no effect on the soft agar growth of BT-474TR cells (Fig. 1b). Thus, BT-474TR cells are trastuzumab-resistant in culture.

We further used nanoparticle tracking analysis (NTA) [22] to characterize EVs emitted by BT-474 cells and observed EV size distribution that is similar to what is often found for EVs emitted by other cell types [22, 23] (Fig. 1c). This distribution did not show a statistically significant difference between BT-474 and BT-474TR cells nor between trastuzumab-treated and untreated cells in the case of both cell lines (Fig. 1c).

We also used electron microscopy to further characterize the particles emitted by BT-474 and BT-474TR cells. EVs were isolated from the cell culture media by ultracentrifugation, a method often used for this purpose [22, 23], in these and further studies. We observed that the morphology and the size of particles emitted by BT-474 cells are similar to those emitted by BT-474TR cells and that these characteristics are not significantly affected by trastuzumab treatment in the case of both cell lines (Fig. 1d). Thus, neither morphology nor the size distribution of EVs emitted by ErbB2-positive breast cancer cells seems to be affected by trastuzumab or by the sensitivity of the cells to this drug.

### Trastuzumab effect on the levels of a mediator of ErbB2 signaling PERP in breast tumor cell-emitted EVs correlates with trastuzumab sensitivity of the cells in culture

Breast epithelial cells are attached in vivo to the ECM [24]. Detachment from the ECM kills them, a phenomenon called anoikis [25]. Anoikis resistance of cancer cells composing 3D tumors and metastases is thought to be critical for tumor progression [20]. We found previously that ErbB2-driven downregulation of a pro-apoptotic protein PERP in breast tumor cells blocks their anoikis [26] and that various ErbB2 antagonists upregulate PERP in these cells [26]. PERP is present in the desmosomes, cell structures mediating cell-to-cell adhesion [27]. PERP kills cells by unknown mechanisms [27]. Of note, amino acid sequence of PERP shows significant similarity to that of the tetraspanins [28], a family of membrane proteins, some of which are present in EVs [29].

**Fig. 2:**
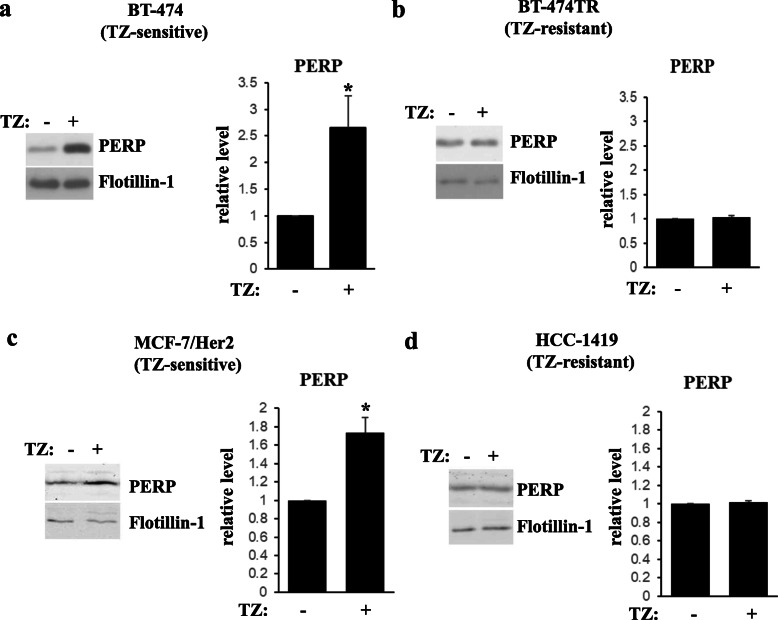
Trastuzumab upregulates PERP in extracellular vesicles emitted by trastuzumab-sensitive but not by trastuzumab-resistant human breast tumor cells. BT-474 (**a**) or BT-474TR (**b**) cells were treated (+) or not (−) with 5 μg/ml trastuzumab (TZ) in 3D culture for 72 h. Extracellular vesicles were isolated from the cell media by ultracentrifugation and assayed for PERP levels by western blot. An extracellular vesicle marker Flotillin-1 is a loading control. Trastuzumab-sensitive MCF7/Her2-18 (**c**) and trastuzumab-resistant HCC-1419 (**d**) human ErbB2-positive breast cancer cells were treated (+) or not (−) with trastuzumab for 48 h (**c**) or 72 h (**d**), and extracellular vesicles were isolated from the cell media as in **a** and analyzed as in **a**. Bar graphs to the right of the western blot images represent quantification of respective bands. PERP levels were normalized by the levels of the loading control. PERP levels (relative level) in the control cells were designated as 1.0. The data represent the average of five (**a**), three (**b**, **c**), and two (**d**) independent experiments, plus SE. *p value was < 0.05

We utilized western blotting to test PERP levels in EVs emitted by BT-474 and BT-474TR cells before and after trastuzumab treatment using an EV marker Flotillin-1 as a loading control. We found that trastuzumab noticeably upregulates PERP in EVs derived from BT-474 cells but not in those emitted by BT-474TR cells (Fig. 2a, b). Trastuzumab also upregulated PERP in EVs emitted by trastuzumab-sensitive breast cancer cells MCF7/Her2-18, a derivative of human breast tumor cells MCF7 producing exogenous ErbB2 [30] (Fig. 2c). In contrast, trastuzumab failed to upregulate PERP in EVs emitted by ErbB2-positive trastuzumab-resistant human breast tumor cells HCC-1419 [18] (Fig. 2d). Thus, trastuzumab upregulates PERP in EVs emitted by trastuzumab-sensitive breast tumor cells but not in those emitted by the trastuzumab-resistant cells.

### Proteomics analysis identifies an additional set of proteins upregulated by trastuzumab in EVs emitted by trastuzumab-sensitive but not by trastuzumab-resistant breast cancer cells

**Fig. 3:**
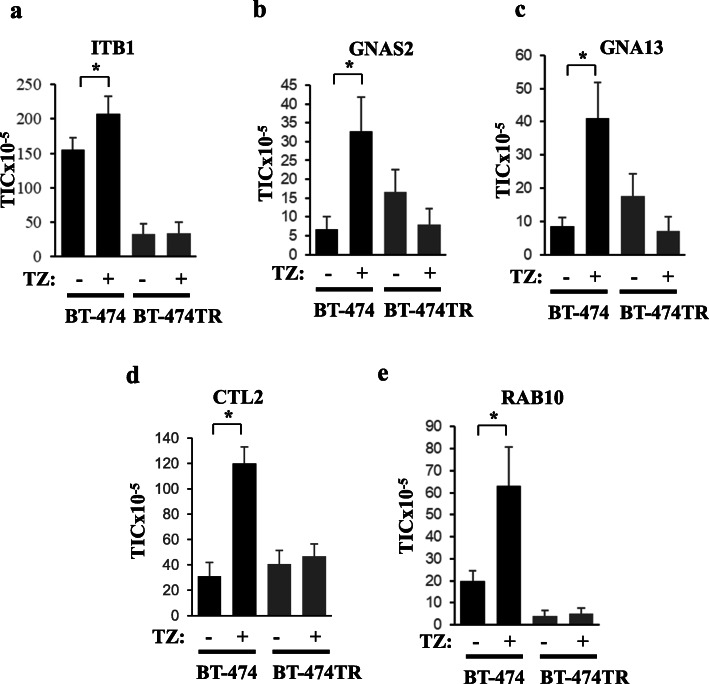
Liquid chromatography-tandem mass spectrometry analysis of proteins in extracellular vesicles emitted by ErbB2-postive human breast tumor cells before and after trastuzumab treatment. BT-474 or BT-474TR cells were treated (+) or not (−) with 5 μg/ml trastuzumab (TZ) in 3D culture for 72 h. Extracellular vesicles were isolated from the cell media by ultracentrifugation. EV protein content was digested by trypsin and subjected to LC-MS/MS. The raw data were matched against the latest human SwissProt protein database using Mascot search engine. Subsequently, label-free TIC quantification was performed by Scaffold Q+ software. About 400 proteins (protein threshold > 95.0%, peptide threshold > 95.0%, and a minimum of two unique peptides per protein) were identified in the EVs. The signal intensities for each of the indicated proteins are expressed in TIC units. The data for ITB1 (**a**), GNAS2 (**b**), GNA13 (**c**), CTL2 (**d**), and RAB10 (**e**) are shown. The data represent the average of three independent experiments plus the SE. *p < 0.05

To test whether, in addition to PERP, levels of other proteins in EVs emitted by breast cancer cells potentially correlate with their trastuzumab response, we used liquid chromatography-tandem mass spectrometry (LC-MS/MS) [22] to perform an initial screen of the proteomes of EVs emitted by BT-474 and BT-474TR cells treated or not with trastuzumab. Analysis of relative protein EV levels was based on label-free protein quantification (Supplementary Table 1). We focused on proteins for which the TIC signals (see the “Methods” section) in EVs emitted by BT474 cells were increased by trastuzumab in at least 3 independent experiments in a statistically significant manner. We found that trastuzumab significantly increased the TIC values for integrin β1 (ITB1), guanine nucleotide-binding protein G subunit alpha isoform short (GNAS2), guanine nucleotide-binding protein subunit alpha-13 (GNA13), choline transporter-like protein 2 (CTL2), and Ras-related protein RAB-10 (RAB10) in EVs emitted by BT-474 cells but not in those produced by BT-474TR cells (Fig. 3). ITB1 is a transmembrane receptor that mediates cell–ECM adhesion [31], GNAS2 [32] and GNA13 [33] are G proteins bound to transmembrane G protein-coupled receptors, and CTL2 is a transmembrane protein that mediates choline transport [34] while RAB10 is a RAS-related small GTPase [35]. Of note, LC-MS/MS did not detect EV-associated PERP in this model which is not uncommon [36, 37] as factors, such as low protein abundance and glycosylation, may limit protein detection in cells and EVs by MS [38, 39]. However, importantly, in agreement with the western blotting data based on the cultured cells (Fig. 2), we repeatedly observed PERP upregulation by western blotting in EVs derived from the blood of breast cancer patients undergoing trastuzumab-based treatments (see Fig. 7).

**Fig. 4:**
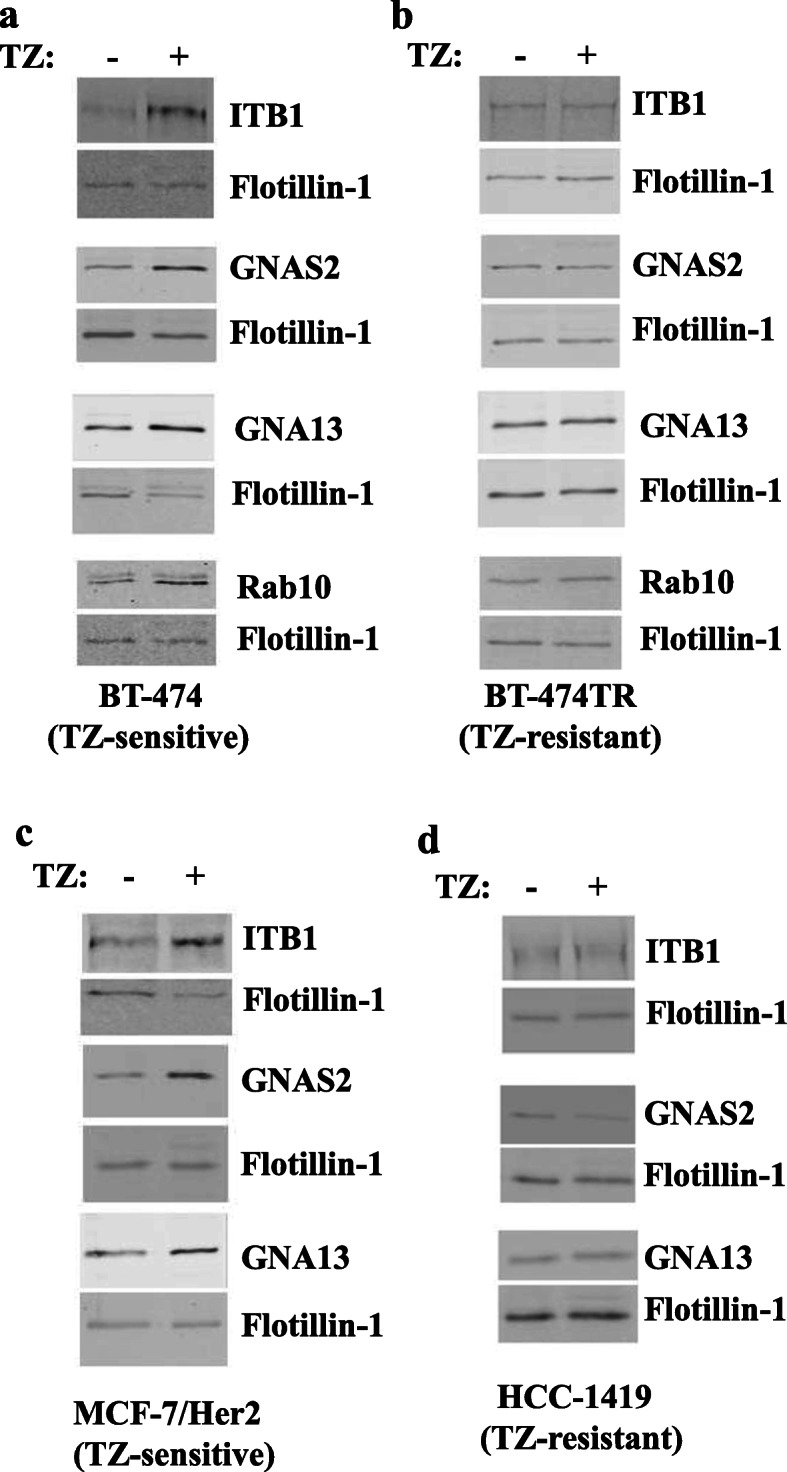
Western blot analysis of protein levels in extracellular vesicles emitted by human ErbB2-positive breast tumor cells. BT-474 (**a**), BT-474TR (**b**), MCF7/Her2-18 (**c**), and HCC-1419 (**d**) human ErbB2-positive breast cancer cell lines were treated (+) or not (−) with trastuzumab (TZ) for 72 h (**a**, **b**, **d**) or 48 h (**c**) in 3D culture, and extracellular vesicles emitted by the cells were isolated by ultracentrifugation and analyzed for the levels of the indicated proteins by western blotting

**Fig. 5:**
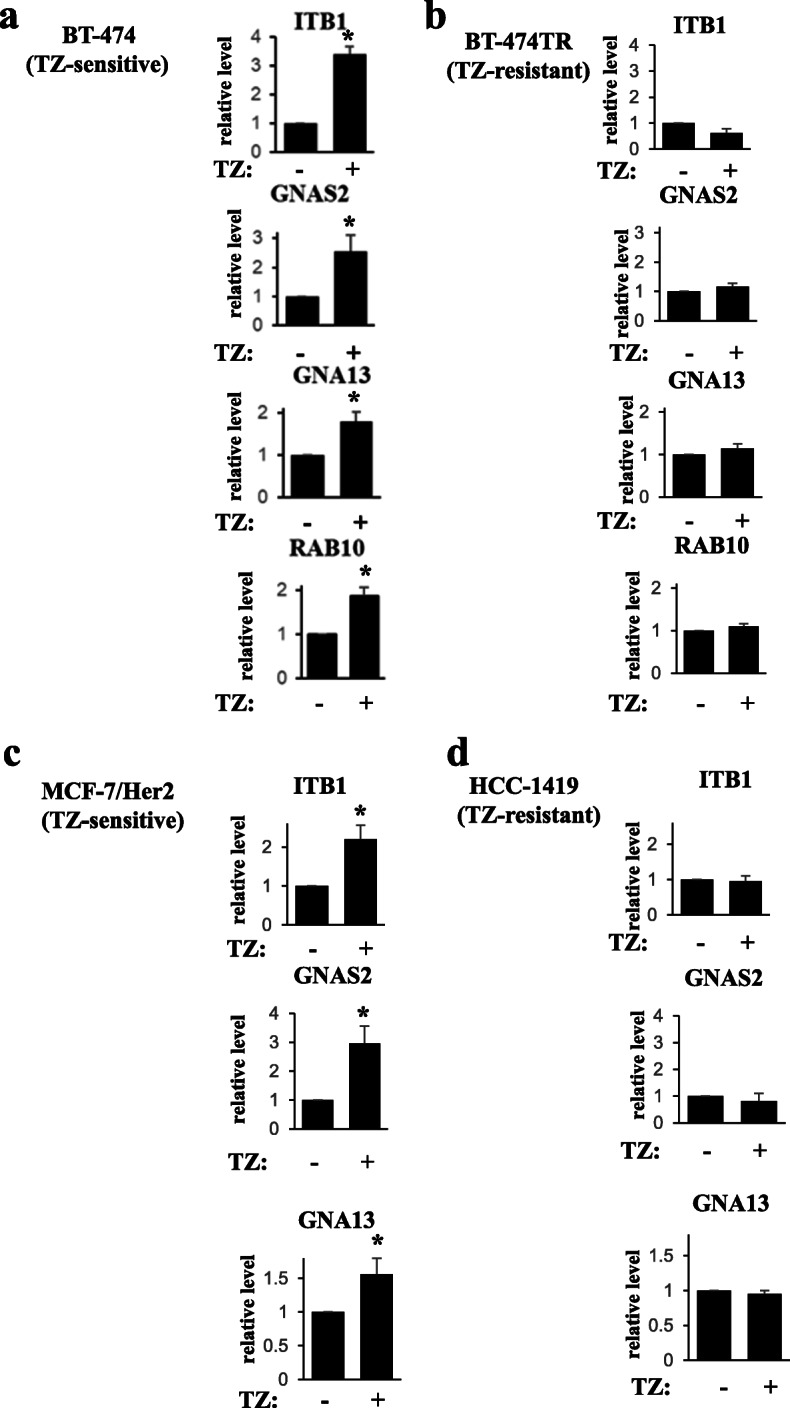
Quantification of the western blot data shown in Fig. 4. The data represent the average of **a** five (ITB1, GNA13, RAB10), six (GNAS2), **b** three, **c** three (ITB1, GNAS2), five (GNA13), and **d** three independent experiments, plus the SE. *p value was < 0.05

**Fig. 6:**
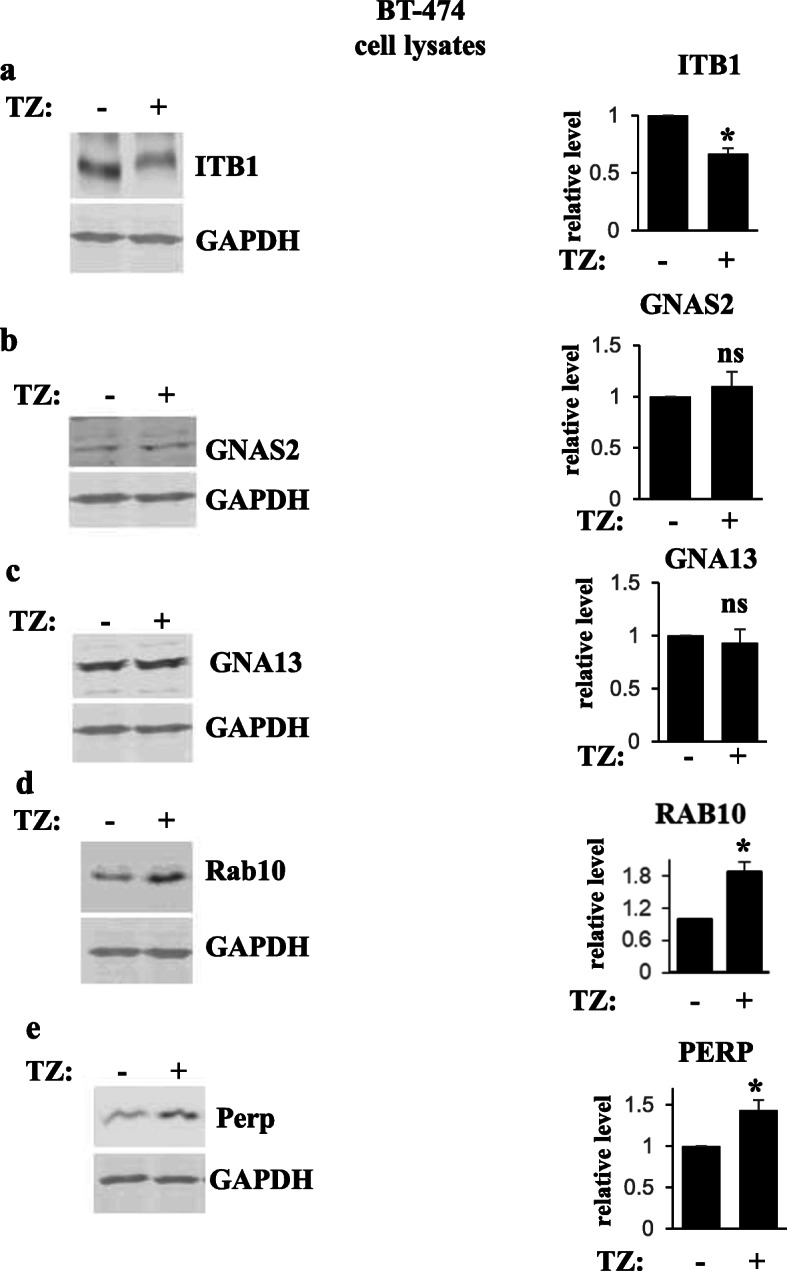
Western blot analysis of protein levels in human ErbB2-positive breast tumor cells. BT-474 were treated (+) or not (−) with trastuzumab (TZ) for 48 h, and cell lysates were assayed for ITB1 (**a**), GNAS2 (**b**), GNA13 (**c**), Rab 10 (**d**), and Perp (**e**) levels by western blotting. GAPDH served as a loading control. Bar graphs to the right of the western blot images represent quantification of respective bands. Protein levels were normalized by the levels of the loading control. Protein levels (relative level) in the control cells were designated as 1.0. The data represent the average of three independent experiments, plus SD. *p value was < 0.05, ns not significant

We have further verified by using western blotting that trastuzumab upregulates ITB1, GNAS2, GNA13, and RAB10 in EVs emitted by BT-474 cells but not by BT-474TR cells (Figs. 4a, b, and 5a, b). In contrast, we have not detected by western blotting reproducible trastuzumab-dependent upregulation of CTL2 in EVs emitted by BT-474 cells (not shown) and did not study this protein further. We also noticed that trastuzumab upregulates ITB1, GNAS2, and GNA13 in EVs emitted by trastuzumab-sensitive breast cancer cells MCF7/Her2-18 [30], but not in EVs emitted by ErbB2-positive trastuzumab-resistant human breast cancer cells HCC-1419 [18] (Figs. 4c, d, and 5c, d). Of note, we have not observed consistent trastuzumab-dependent upregulation of RAB10 in EVs emitted by MCF7/Her2-18 cells (not shown). Interestingly, unlike the case with EVs, trastuzumab did not upregulate all of the indicated proteins in the breast cancer cells themselves: the drug upregulated RAB10 and PERP in BT-474 cells (Fig. 6d, e) but downregulated ITB1 (Fig. 6a) and did not change the levels of GNAS2 and GNA13 in the cells (Fig. 6b, c). In summary, we have demonstrated that trastuzumab upregulates PERP, ITB1, GNAS2, and GNA13 in EVs emitted by two different trastuzumab-sensitive breast cancer cell lines but not in EVs derived from two different trastuzumab-resistant breast cancer cell lines (Figs. 2, 3, 4, and 5).

### Levels of EV proteins correlate with breast cancer patient trastuzumab responses in a pilot patient cohort

**Fig. 7:**
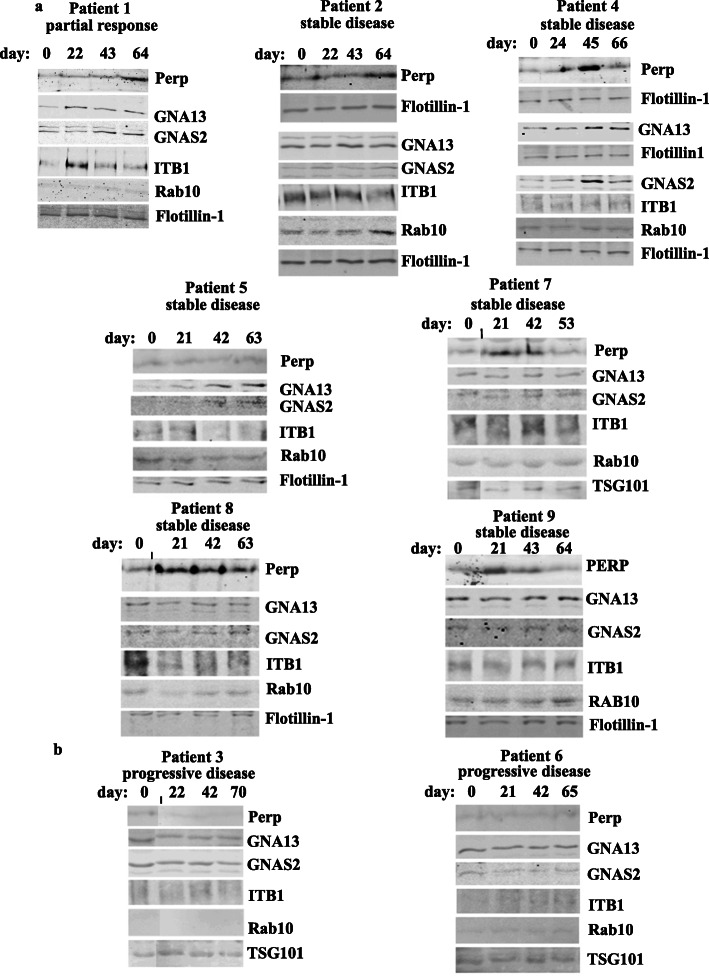
PERP and/or GNAS2 and/or GNA13 and/or ITB1 and/or RAB10 are upregulated in the blood extracellular vesicles of patients with ErbB2-positive metastatic breast cancer that benefited from trastuzumab-based therapies but not in those derived from patients that failed these treatments. Blood was collected from the indicated patients on the indicated days during trastuzumab (TZ)-based therapies. The day of the first blood collection was designated as “day 0.” Normally, blood was collected 1 day prior to the therapy. The extracellular vesicles were isolated from patients’ blood by ultracentrifugation and tested for the levels of the indicated proteins by western blotting. Extracellular vesicle markers Flotillin-1 and TSG101 are loading controls. Patients’ metastatic disease status (shown above each blot) was assessed based on the comparison of their CT scans performed as closely as possible to the dates of the first and the last blood collection. Data for patients showing partial response or stable disease (**a**) or those with progressive disease (**b**) are shown

We further tested whether it is feasible to study EV proteins described above as potential biomarkers of metastatic breast cancer trastuzumab response in breast cancer patients. To this end, we selected a pilot cohort of 10 patients of 34–60 years of age with metastatic ErbB2-positive breast cancer, nine of which were undergoing trastuzumab-based therapies at the QEII Health Centre in Halifax, NS, during the study. One patient (patient 3) had failed trastuzumab-based therapy prior to the study and was receiving T-DM1 (a drug that acts on tumor cells via different mechanisms than trastuzumab [5]) during the study. Since the patient had already failed trastuzumab-based treatments, we reasoned that respective samples might represent good negative controls for the study, i.e., if levels of respective blood EV proteins indeed correlate with patients’ trastuzumab response, we expected these levels not to increase with time in EVs derived from the patient (and this turned out to be the case, see Figs. 7b and 9a). Of note, all patients, except for patient 1, were on trastuzumab prior to the study while patient 1 began receiving trastuzumab during the study. We later excluded one of the trastuzumab-treated patients from the study as the data derived from her EVs were hard to interpret for technical reasons. The status of the lesions in the remaining patients was monitored by CT using Response Evaluation Criteria in Solid Tumors [9] (Supplementary fig. 1). Each patient had four consecutive blood samples drawn 3 weeks apart.

**Fig. 8:**
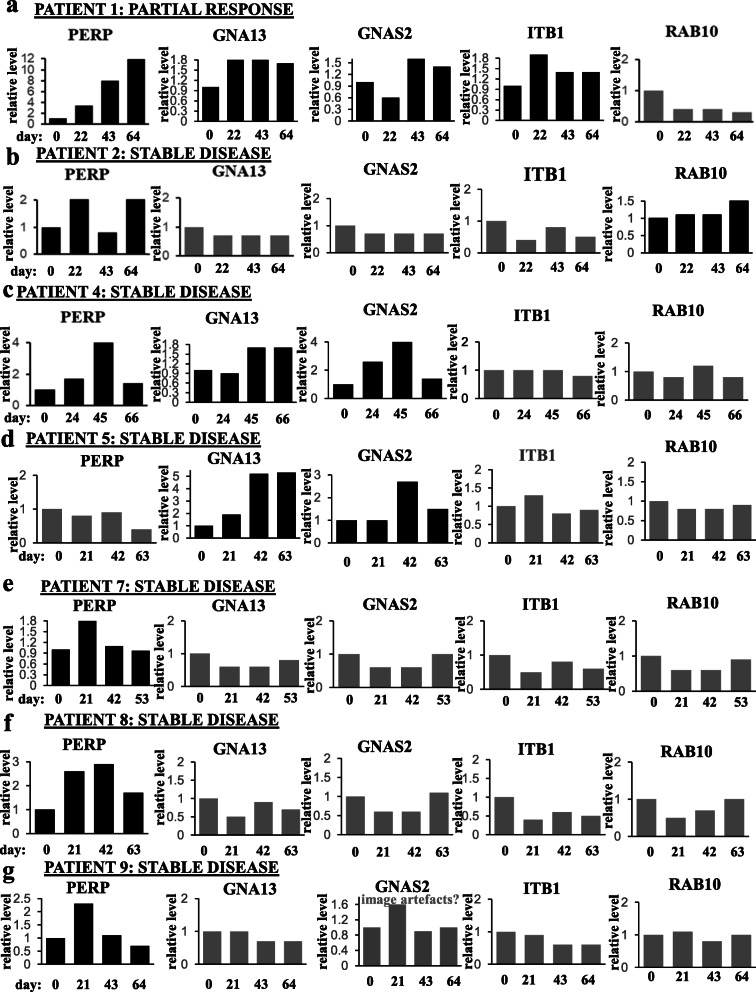
Quantification of the western blot data for patients that benefited from trastuzumab-based therapies shown in Fig. 7a. The data for patient 1 (**a**), patient 2 (**b**), patient 4 (**c**), patient 5 (**d**), patient 7 (**e**), patient 8 (**f**), and patient 9 (**g**) are shown. Note that statistical analysis of the data was not possible as blood was collected from each patient only once at each time point. In the case of patient 9, an apparent increase in the GNAS2 level on day 21 likely reflects artifacts (“image artifacts?”) on the respective image (see Fig. 7)

**Fig. 9:**
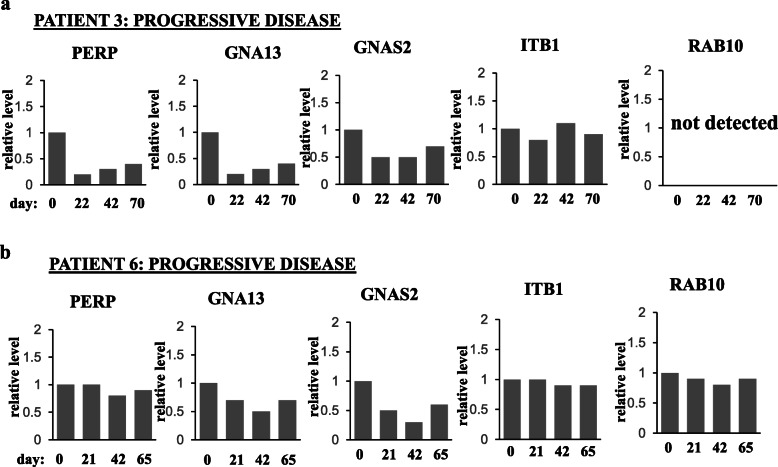
Quantification of the western blot data for patients that did not benefit from trastuzumab-based therapies shown in Fig. 7b. The data for patient 3 (**a**) and patient 6 (**b**) are shown. Note that statistical analysis of the data was not possible as blood was collected from each patient only once at each time point

We utilized western blotting to test PERP, ITB1, GNAS2, GNA13, and RAB10 levels in the patients’ blood EVs using EV markers Flotillin-1 or TSG101 as loading controls (Fig. 7). We noticed that either PERP, GNA13, GNAS2, and ITB1 (patient 1), or PERP and RAB10 (patient 2) or PERP, GNA13, and GNAS2 (patient 4), or GNA13 and GNAS2 (patient 5) or PERP alone (patients 7, 8, and 9) were upregulated in the blood EVs during the treatment when patients benefited from the therapy, i.e., showed partial response or stable disease (when the size of the lesions does not change significantly [4]) (Fig. 7a). Importantly, none of these proteins was upregulated in the course of the treatment in EVs of patient 3 who failed trastuzumab treatment prior to the study or in EVs of patient 6 who did not respond to the therapy and had progressive disease (Fig. 7b). The latter two results support the notion that the upregulation of the proteins in EVs derived from the trastuzumab-sensitive patients is the consequence of trastuzumab response of their tumors, rather than that of accidental time-dependent fluctuations of these levels. The data shown in Fig. 7a and b are quantified in Figs. 8 and 9, respectively. Of note, statistical analysis of the quantitative data was not possible in these cases by definition as the blood was collected from each patient only once at each time point. Possible reasons why all five proteins were not upregulated in all cases when patients did benefit from the therapy are that the patients received different trastuzumab-based treatment regimens (see Supplementary fig. 1) or that a protein was upregulated at time points not captured by the study. Thus, our data underscore the need for monitoring several EV proteins during the therapy, rather than one, for assessing trastuzumab efficacy. Of note, of those patients whose EV protein levels correlated with TZ response (Fig. 7), patients 4, 5, 6, 7, 8, and 9 were on trastuzumab prior to the study while patient 1 began receiving trastuzumab during the study. Thus, the indicated protein levels seem to correlate with patients’ trastuzumab response regardless of whether the patients are “treatment-naive” at the study onset.

In summary, we have identified proteins whose upregulation in EVs derived from different breast tumor cell lines correlates with tumor cell trastuzumab sensitivity. Moreover, we have demonstrated that further studies of these proteins as potential biomarkers of breast cancer patient trastuzumab response are feasible.

## Discussion

We found in this study that trastuzumab upregulates a set of proteins in EVs emitted by trastuzumab-sensitive but not by trastuzumab-resistant ErbB2-positive breast cancer cells in culture. In addition, we observed that upregulation of these proteins in the blood-derived EVs seems to correlate with clinical benefit that a pilot cohort of breast cancer patients with metastatic ErbB2-positive tumors received from trastuzumab-based therapies. Collectively, these data indicate that further examination of these proteins as potential biomarkers of breast cancer patient trastuzumab response is feasible.

One of the indicated proteins is PERP (Figs. 2, 7, and 8) that, according to our previous studies, represents an important mediator of ErbB2 signaling: we observed in the past that ErbB2-driven downregulation of PERP is critical for 3D growth of breast cancer cells [26]. Other potential biomarkers of breast cancer trastuzumab response are proteins GNAS2, GNA13, RAB10, and ITB1 (Figs. 3, 4, 5, 8, and 9) that we discovered via proteomics-based analysis (Fig. 3). Whether or not any of the latter proteins contribute to ErbB2-driven breast cancer is an important question for our further studies.

Interestingly, we observed that neither trastuzumab nor acquisition of resistance to this drug affects the number or the size of the nanoparticles emitted by breast cancer cells (Fig. 1c). Thus, trastuzumab-dependent upregulation of the indicated EV proteins does not seem to occur due to an increased emission of a particular EV subtype. Also notably, even though trastuzumab upregulated PERP, GNAS2, GNA13, RAB10, and ITB1 in EVs emitted by trastuzumab-sensitive breast cancer cells (Figs. 2, 3, and 4), the drug did not have this effect on all of these proteins in the cells themselves: trastuzumab downregulated ITB1, upregulated RAB10 and PERP, and had no effect on GNAS2 and GNA13 in the indicated cells (Fig. 6). Understanding the mechanisms by which trastuzumab upregulates the indicated proteins in EVs represents a promising direction for our future work.

Importantly, we observed that PERP, ITB1, GNAS2, and GNA13 are upregulated by trastuzumab in ErbB2-positive human breast cancer cells BT-474 and MCF-7/Her2-18 (Figs. 2, 3, and 4) which indicates that the effect of trastuzumab on these proteins is not unique to one cell line. Moreover, these observations do not seem to be limited to tissue culture as we observed that these proteins are upregulated in various combinations in breast cancer patient blood-derived EVs during trastuzumab-based therapies in those cases when the patients benefited from the therapy (Figs. 7 and 8). Of note, we detected trastuzumab-dependent upregulation of a protein RAB10 in EVs derived from BT-474 cells (Fig. 4a) but not in those emitted by MCF-7/Her218 cells (not shown). However, the effect of trastuzumab on RAB10 is not unique to one model system as we also observed RAB10 upregulation in EVs derived from the blood of one of the breast cancer patients undergoing trastuzumab-based therapy that did benefit from the treatment (Figs. 7 and 8). Thus, even though upregulation of RAB10 was detected by us more rarely than that of other proteins, RAB10 still appears to be a worthy candidate for further studies.

Our data indicate that PERP, GNA13, GNAS2, ITB1, and RAB10 are upregulated in EVs derived from trastuzumab-sensitive but not from trastuzumab-resistant breast cancer cells in culture. Moreover, at least one of these proteins was upregulated in EVs derived from the blood of all patients with metastatic breast cancer recruited for the study that did benefit from trastuzumab-based therapies (Fig. 7a and 8). In contrast, none of these proteins was upregulated in EVs of the patients that did not benefit from the therapy (Fig. 7b and 9). The latter results are consistent with the notion that upregulation of the EV proteins in the blood of trastuzumab-sensitive patients is the consequence of response of their tumors to trastuzumab-based treatments, rather than that of time-dependent fluctuations of the levels of these proteins. Collectively, our data indicate that the proteins in question are promising candidates for the biomarkers of breast cancer trastuzumab response.

Possible reasons why all of the proteins in question were not upregulated in all cases when patients benefited from the therapy or were upregulated transiently in some cases are that patients’ trastuzumab-based treatment regimens were not the same in all cases (see supplemental fig. 1) or that some of these proteins were upregulated at time points not captured by the study. It is also possible that the effects of trastuzumab on some of these proteins vary between patients. Investigating how treatment regimens and perhaps other clinical parameters affect EV levels of the proteins in question using a larger patient cohort, where these correlations could be statistically significant, represents an important goal of our future studies. Our data indicate that accurate assessment of trastuzumab efficacy might require simultaneous monitoring of several EV proteins during the therapy, rather than one, and/or that these proteins might need to be monitored more frequently than was done in our study. In addition, ELISAs or antibody arrays could potentially detect these proteins more robustly in a clinical setting than western blotting.

It is noteworthy that of those patients whose EV protein levels correlated with trastuzumab response (Figs. 7 and 8), patients 4, 5, 6, 7, 8, and 9 were receiving trastuzumab prior to the study whereas patient 1 began receiving the drug during the study. Thus, the indicated protein levels seemed to correlate with patients’ trastuzumab response regardless of whether the patients were “treatment-naive” at the beginning of the study.

How could patients benefit from the use of such biomarkers? Trastuzumab is an expensive drug [6] that can have significant side effects, such as cardiotoxicity [7]. Availability of biomarkers indicating that patient tumors are no longer responding to trastuzumab could allow oncologists to detect this lack of response by imaging studies earlier than routinely planned and switch patients to the second-line drug T-DM1 sooner or perhaps even perform the indicated switch without waiting for imaging studies. The patient could then avoid unwarranted treatment with the ineffective and expensive drug in a timely manner and benefit from the early switch to T-DM1 earlier than is presently possible [5]. Moreover, blastic or mixed blastic/lytic bone-only breast cancer metastases are considered non-measurable lesions and their response to trastuzumab is not easily measured by imaging studies alone [9]. Hence, availability of a biomarker indicating whether such lesions respond to trastuzumab-based therapies could significantly facilitate further treatment decisions. Evaluating the indicated proteins as potential biomarkers of metastatic ErbB2-positive breast cancer response to trastuzumab-based therapies in a large patient cohort represents an important future direction of our research.

## Conclusions

Our study indicates that levels of proteins PERP, GNAS2, GNA13, ITB1, and RAB10 in EVs emitted by cultured breast cancer cells correlate with sensitivity of these cells to trastuzumab. Moreover, at least one of these proteins was upregulated in EVs derived from the blood of a pilot cohort of metastatic breast cancer patients during trastuzumab-based therapies in those cases when patients benefited from these treatments but not in those derived from patients whose tumors were resistant to these therapies. Thus, further studies of the indicated proteins as potential biomarkers of breast cancer patient trastuzumab response are feasible.

## Supplementary information

**Additional file 1: Supplementary Table 1.** Liquid chromatography-tandem mass spectrometry analysis of proteins in extracellular vesicles emitted by BT-474 or BT-474TR cells before and after trastuzumab treatment. BT-474 or BT-474TR cells were treated (+) or not (−) with 5 μg/ml trastuzumab (TZ) in 3D culture for 72 h. Extracellular vesicles were isolated from the conditioned media by ultracentrifugation. EV proteins were digested by trypsin and analyzed by LC-MS/MS. The raw data were matched against the latest human SwissProt protein database by use of the Mascot search engine. Label-free TIC quantification was further performed by Scaffold Q+ software. Signal intensities for each of the indicated EV proteins are expressed in TIC units (protein threshold >95.0%, peptide threshold >95.0%, a minimum of two unique peptides identified per protein). The data for five independent experiments are shown.

**Additional file 2: Supplementary Fig. 1** The status of metastatic disease of breast cancer patients with ErbB2-positive metastatic breast cancer whose blood was used in the study. (**a**) The disease status was determined in the case of each patient based on the comparison of CT scan 2 with CT scan 1 using Response Evaluation Criteria in Solid Tumors. Treatment regimens received by each patient during the time period when the blood was collected are indicated. The dates of the CT scans are indicated. (**b**) CT scans of patient 1 performed on the indicated dates are shown as examples of imaging studies used by us. Comparison of the scans shows that patient’s axillary lymph node was smaller on 07/23/18 than on 05/14/18.

## Data Availability

The datasets used and/or analyzed during the current study are available from the corresponding author on reasonable request.

## Abbreviations

EV: Extracellular vesicle
MS: Mass spectroscopy
LC-MS/MS: Liquid chromatography-tandem mass spectrometry
PBS: Phosphate-buffered saline
NTA: Nanoparticle tracking analysis
TIC: Total ion count
CT: Computer tomography
RECIST: Response Evaluation Criteria in Solid Tumors
