# Correction to: Trastuzumab-induced upregulation of a protein set in extracellular vesicles emitted by ErbB2-positive breast cancer cells correlates with their trastuzumab sensitivity

**DOI:** 10.1186/s13058-020-01358-8

**Published:** 2020-10-22

**Authors:** Arik Drucker, Byong Hoon Yoo, Iman Aftab Khan, Dongsic Choi, Laura Montermini, Xiaoyang Liu, Sanja Jovanovic, Tallal Younis, Kirill V. Rosen

**Affiliations:** 1 Department of Medicine, Dalhousie University, Halifax, NS Canada; 2 Departments of Pediatrics & Biochemistry and Molecular Biology, Dalhousie University, Halifax, NS Canada; 3 Research Institute of the McGill University Health Centre, Glen Site, McGill University, Montreal, QC Canada; 4 Atlantic Research Centre, Rm C-304, CRC, 5849 University Avenue, PO Box 15000, Halifax, NS B3H 4R2 Canada

**Correction to: Breast Cancer Res 22, 105 (2020)**

**https://doi.org/10.1186/s13058-020-01342-2**

Following publication of the original article [1], the authors identified an error in the author name of Arik Drucker.

The incorrect author name is: Arik Durcker.

The correct author name is: Arik Drucker.

The author group has been updated above and the original article [1] has been corrected.
