# Supplementary material for: Trastuzumab-induced upregulation of a protein set in extracellular vesicles emitted by ErbB2-positive breast cancer cells correlates with their trastuzumab sensitivity
Source: Breast Cancer Res. 2020 Oct 6;22:105. doi: 10.1186/s13058-020-01342-2 (PMC7541295; doi:10.1186/s13058-020-01342-2)
Supplement: Supplementary file 2 — Additional file 2: Supplementary Fig. 1 The status of metastatic disease of breast cancer patients with ErbB2-positive metastatic breast cancer whose blood was used in the study. (a) The disease status was determined in the case of each patient based on the comparison of CT scan 2 with CT scan 1 using Response Evaluation Criteria in Solid Tumors. Treatment regimens received by each patient during the time period when the blood was collected are indicated. The dates of the CT scans are indicated. (b) CT scans of patient 1 performed on the indicated dates are shown as examples of imaging studies used by us. Comparison of the scans shows that patient’s axillary lymph node was smaller on 07/23/18 than on 05/14/18. [file 13058_2020_1342_MOESM2_ESM.ppt]

## Slide 1
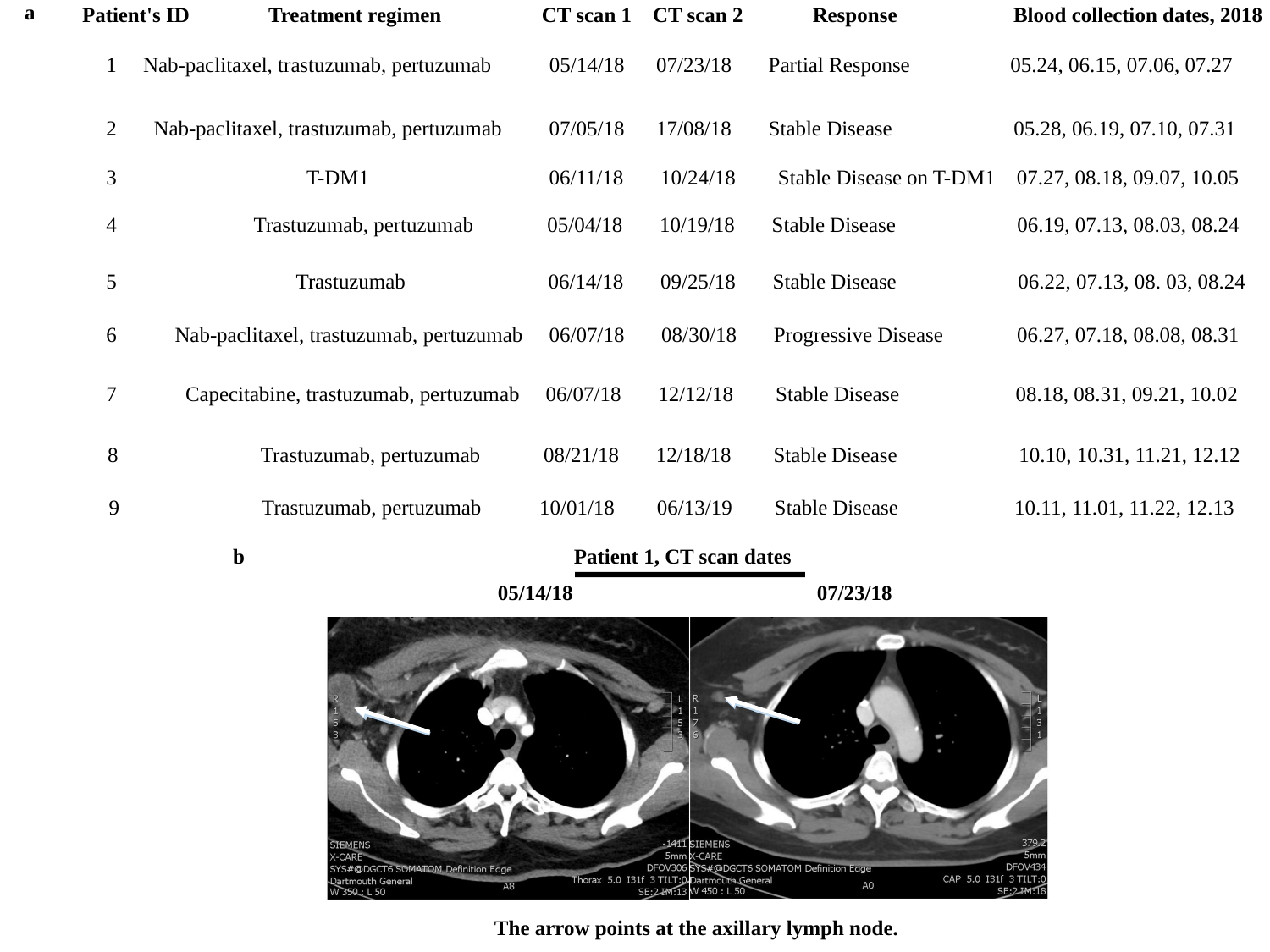

| a |
| --- |
Patient's ID Treatment regimen CT scan 1 CT scan 2 Response Blood collection dates, 2018
1 Nab-paclitaxel, trastuzumab, pertuzumab 05/14/18 07/23/18 Partial Response 05.24, 06.15, 07.06, 07.27
2 Nab-paclitaxel, trastuzumab, pertuzumab 07/05/18 17/08/18 Stable Disease 05.28, 06.19, 07.10, 07.31
3 T-DM1 06/11/18 10/24/18 Stable Disease on T-DM1 07.27, 08.18, 09.07, 10.05
4 Trastuzumab, pertuzumab 05/04/18 10/19/18 Stable Disease 06.19, 07.13, 08.03, 08.24
5 Trastuzumab 06/14/18 09/25/18 Stable Disease 06.22, 07.13, 08. 03, 08.24
6 Nab-paclitaxel, trastuzumab, pertuzumab 06/07/18 08/30/18 Progressive Disease 06.27, 07.18, 08.08, 08.31
7 Capecitabine, trastuzumab, pertuzumab 06/07/18 12/12/18 Stable Disease 08.18, 08.31, 09.21, 10.02
8 Trastuzumab, pertuzumab 08/21/18 12/18/18 Stable Disease 10.10, 10.31, 11.21, 12.12
9 Trastuzumab, pertuzumab 10/01/18 06/13/19 Stable Disease 10.11, 11.01, 11.22, 12.13
Patient 1, CT scan dates
| b |
| --- |
05/14/18
07/23/18
The arrow points at the axillary lymph node.
